# Binder-Centered Design of Sustainable Liquid Metal Composites for Adaptive Soft Energy Storage Systems: A Framework-Driven Perspective Review

**DOI:** 10.3390/polym18131650

**Published:** 2026-07-02

**Authors:** Elahe Parvini, Abdollah Hajalilou

**Affiliations:** 1 Institute of Systems and Robotics, Department of Electrical Engineering, University of Coimbra, 3030-290 Coimbra, Portugal; 2 CENIMAT|i3N, Department of Materials Science, School of Science and Technology, Campus de Caparica, NOVA University Lisbon, 2829-516 Caparica, Portugal

**Keywords:** sustainable liquid metal composites, polymer binders, soft energy storage, self-healing materials, printable electronics, interfacial engineering

## Abstract

Gallium (Ga)-based liquid metal (LM) composites, particularly those based on eutectic gallium–indium (EGaIn) and related alloys, have emerged as a promising materials platform for soft and deformable energy storage owing to their unique combination of metallic conductivity, fluidic deformability, and adaptive interfaces. Despite rapid advances in LM-enabled devices, binders remain insufficiently understood and are still commonly regarded as passive structural components. Here, we present a comprehensive binder-centered perspective for LM composites, establishing the binder as a key regulator of electro-chemo-mechanical coupling, interfacial stability, transport behavior, and processability in soft energy systems. We show that tailored binder chemistries in Ga-based LM systems—including stretchable batteries, printable conductors, and soft electrochemical devices—govern LM droplet dispersion, suppress coalescence and leakage, and preserve conductive percolation under large deformation, while enabling room-temperature fabrication and printability through rheological regulation and interfacial wetting. Beyond mechanical confinement, emerging binder functionalities—including dynamic bonding, supramolecular interactions, ionically conductive networks, and reversible polymer architectures—enable self-healing interfaces, adaptive transport pathways, and robust adhesion in deformable devices. By integrating recent advances in stretchable batteries, flexible supercapacitors, printable electronics, and multifunctional soft energy systems, we establish a unified multiscale framework linking binder molecular design to device-level electrochemical and mechanical performance. We further discuss sustainability and manufacturing considerations, including recyclable polymer networks, low-temperature fabrication, and scalable processing strategies. Finally, we outline current challenges and future opportunities toward programmable binder systems with tunable viscoelasticity, interfacial reactivity, and adaptive functionality. This Review establishes binder-centered engineering as a key pathway for transforming LM composites from proof-of-concept materials into resilient, manufacturable, and multifunctional soft energy technologies for wearable, stretchable, and biointegrated electronics.

## 1. Introduction

The rapid emergence of soft and deformable electronic systems—including wearable sensors, electronic skins, and biointegrated platforms—has created an urgent demand for energy storage technologies capable of maintaining stable electrochemical performance under mechanical deformation [1,2,3,4,5]. Conventional batteries, based on rigid electrode architectures and brittle interfacial contacts, are fundamentally incompatible with such requirements and typically suffer from fracture, delamination, and loss of electrical continuity under strain [1,4,5,6]. Developing intrinsically compliant energy storage systems that can sustain coupled electrochemical and mechanical loading therefore remains a central challenge in next-generation electronics [5,6,7].

Among emerging material platforms, Ga-based LMs—particularly Ga-based alloys such as EGaIn—have attracted significant attention due to their unique combination of metallic conductivity, fluidity, and near-zero elastic modulus [8,9,10,11,12]. Unlike conventional rigid conductors, LM phases can undergo large, reversible deformation without fracture, enabling continuous conductive pathways under bending, stretching, and twisting [9,10,11,13]. In addition, the spontaneous formation of a thin native oxide skin stabilizes LM droplets and enables adhesion to surrounding materials, while introducing dynamic interfacial behavior governed by oxidation, wetting transitions, and mechanical perturbations [10,12,14,15].

Despite these advantages, integrating LMs into electrochemical systems remains challenging. The high surface tension of Ga-based LMs (~500–700 mN/m depending on composition and oxidation state) drives droplet spheroidization and coalescence, hindering uniform dispersion within composite matrices and promoting phase separation under mechanical perturbation [8,12,16,17,18]. Under repeated deformation, such behavior can disrupt conductive percolation networks and compromise device reliability [12,13,16,18,19]. In parallel, LM-based systems exhibit inherently dynamic interfacial behavior, including oxide-layer evolution, wetting changes, and compositional redistribution during electrochemical cycling, all of which strongly influence charge transfer and long-term stability [14,15,20,21,22].

In conventional battery electrodes, binders are typically treated as passive components that provide adhesion between active materials and current collectors while maintaining structural cohesion [23,24,25,26]. Widely used systems such as poly(vinylidene fluoride) (PVDF) and carboxymethyl cellulose (CMC) are optimized primarily for mechanical integrity and slurry processability, assuming static interfaces and rigid architectures [23,24,27,28]. This paradigm becomes inadequate in LM composites, where a fluid metallic phase introduces fundamentally different structural and interfacial requirements. In these systems, the binder must actively regulate droplet dispersion, suppress coalescence and leakage, and preserve conductive percolation under deformation, rather than functioning solely as an adhesive matrix [16,19,29].

Recent studies demonstrate that binder chemistry plays a decisive role in stabilizing LM-containing systems. Polymer binders incorporating dynamic noncovalent interactions—such as hydrogen bonding, ionic interactions, and ion–dipole coupling—enable stress dissipation, structural recovery, and improved electrochemical stability [7,15,29,30,31]. These dynamic networks allow composites to accommodate deformation while maintaining electrical connectivity, indicating that binder design directly governs electro-chemo-mechanical coupling in LM systems. More broadly, advances in functional binders show that polymer matrices can influence ion transport, electronic conductivity, and electrode–electrolyte interfacial behavior in advanced electrochemical systems [24,32,33]. Similar stabilization principles are also important in LM-based thermal interface materials (TIMs), where droplet confinement and leakage suppression critically influence thermal and mechanical reliability [34,35].

Binder design is equally critical for enabling the processability and manufacturability of LM composites. Due to their high surface tension and low viscosity, bare LMs are difficult to pattern and lack shape stability during deposition [8,12,18]. Polymer matrices and composite architectures are therefore employed to tune rheological behavior, stabilize LM dispersions, and enable additive manufacturing techniques such as direct ink writing and extrusion printing, spray coating, and jet printing [36,37,38,39]. In biphasic LM composites, elastomeric binders and conductive fillers create viscoelastic inks that flow under shear but retain their geometry after deposition, enabling stretchable conductive structures at room temperature [27,37,38].

**Figure 1:**
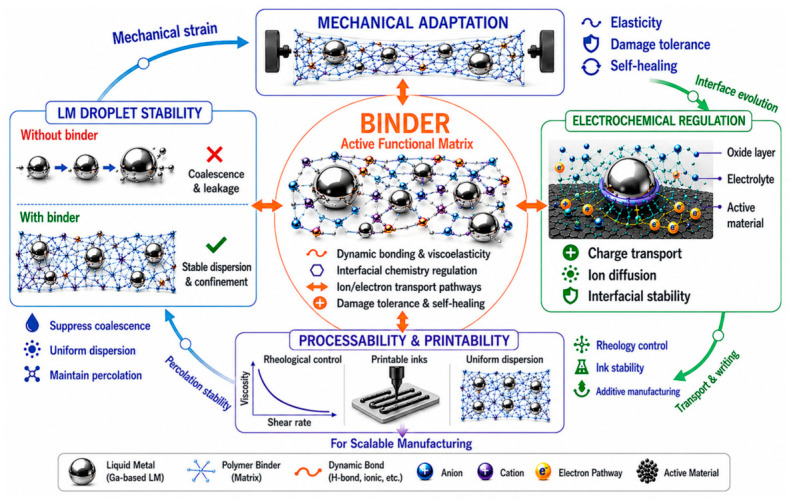
Binder-centered framework for LM composites in soft energy storage. The binder functions as an active matrix that simultaneously regulates mechanical behavior, electrochemical interfaces, and manufacturing processes, thereby enabling stable, scalable, and high-performance soft energy systems.

At the device level, binder-mediated LM composites have enabled new classes of soft energy systems. In stretchable batteries, composite matrices confine LM droplets and prevent uncontrolled redistribution during deformation, preserving electrode integrity and electrochemical functionality [40,41,42,43,44]. In integrated soft electronic systems, elastomeric binders provide interlayer adhesion and mechanical compatibility, enabling fully printed, multilayer devices with enhanced resilience and recyclability [41,45]. These results highlight that device performance is determined not only by the intrinsic properties of the LM, but also by how effectively the binder stabilizes and integrates the composite structure across multiple length scales. As illustrated in Figure 1, binder-centered engineering enables the coupling of LM droplet stabilization, electrochemical interface regulation, mechanical adaptability, and processability, thereby governing the electro-chemo-mechanical behavior of soft LM-based energy systems across multiple length scales.

Previous reviews have provided valuable overviews of liquid metal composites, stretchable electronics, printable conductors, and functional binder chemistries. However, these studies have generally addressed liquid metal behavior, device performance, and polymer design as separate topics. In contrast, the present review adopts a binder-centered framework that considers the binder as an active regulator of LM droplet stabilization, interfacial evolution, transport behavior, processability, and electro–chemo–mechanical performance. By integrating these interconnected aspects, this perspective provides a unified design framework for adaptive soft energy storage systems.

Despite significant progress, a unified framework linking binder molecular design with LM droplet dynamics, interfacial evolution, and electrochemical performance under deformation remains lacking [30,32,33]. This gap limits the rational design of LM composites and hinders their translation into scalable soft energy storage technologies.

In this Review, we present a binder-centered perspective on Ga-based LM composites for soft energy storage. We establish the binder as a key design element that couples mechanical confinement, interfacial stability, electrochemical functionality, and processability. By integrating insights from polymer chemistry, interfacial science, and electrochemical engineering, we develop a unified framework linking binder molecular design to device-level performance under coupled electro-chemo-mechanical conditions.

## 2. From Passive Adhesion to Active Function: Redefining the Role of Binders

**Figure 2:**
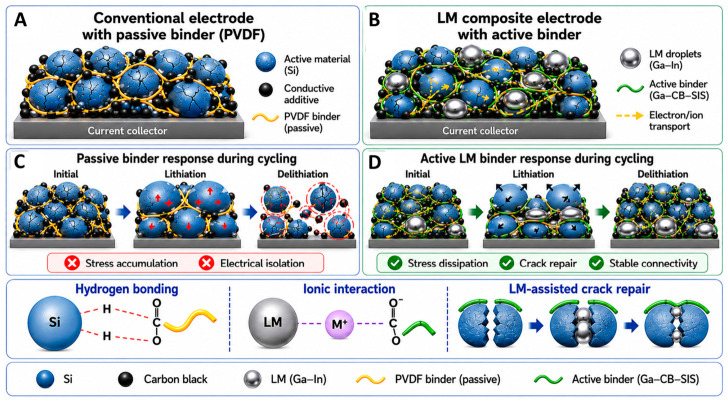
Transition from passive to active binder behavior in liquid metal composite electrodes. (**A**) Conventional electrode with a passive binder (e.g., PVDF), where the binder primarily provides adhesion and cohesion, while electron transport is maintained through the conductive additive network. (**B**) LM composite electrode with an active binder, where the polymer matrix confines LM droplets, stabilizes interfaces, enables self-healing, and maintains coupled electron/ion transport under deformation. (**C**) Schematic illustration of the mechanical and electrical degradation of passive binder systems during lithiation/delithiation cycling, resulting in stress accumulation and electrical isolation. (**D**) Active binder regulation enabled by LM incorporation, promoting stress dissipation, crack repair, interfacial stabilization, and reliable electrical connectivity. The lower panel summarizes representative interfacial mechanisms associated with active binder behavior, including hydrogen bonding, ionic interactions, and LM-assisted crack repair.

In conventional battery electrodes, binders are typically regarded as passive yet essential components that maintain adhesion between active materials and current collectors, preserve electrode cohesion, and enable slurry-based processing [23,24,27,46,47]. Industrial systems such as poly(vinylidene fluoride) (PVDF) for cathodes and carboxymethyl cellulose (CMC)-based binders for anodes reflect a design paradigm focused primarily on mechanical integrity and processability rather than electrochemical functionality [23,24,27,28,46,47]. While effective for rigid particulate electrodes, this framework implicitly assumes static interfaces and mechanically stable architectures. As illustrated in Figure 2A, conventional electrodes consist of active particles, conductive additives, and a polymer binder that forms sparse interparticle connections. In this configuration, electron transport is sustained by the conductive additive network, while the binder contributes negligibly to electronic or ionic transport and does not actively regulate interfacial processes [23,24,27,46]. Consequently, electrochemical performance depends on stable particle–particle contact, making the system vulnerable to mechanical degradation, particle isolation, and loss of conductive pathways under stress [24,46,47]. However, these assumptions break down in LM composite systems, where the presence of a fluidic metallic phase introduces fundamentally different physical and interfacial behavior. Ga-based LMs exhibit metallic conductivity, fluidity, and near-zero elastic modulus, enabling stable electrical performance under large deformation and making them attractive for stretchable and wearable electronics [8,9,10,11,12]. However, their high surface tension drives droplet spheroidization and resists uniform dispersion, posing a major challenge for achieving stable composite microstructures and printable inks [8,9,16,17]. Recent studies on biphasic EGaIn–elastomer composites further demonstrated that elastomeric binder networks can kinetically stabilize LM dispersions through viscoelastic confinement and suppression of droplet migration during repeated deformation [48,49,50,51]. As a result, insufficiently regulated systems may exhibit droplet coalescence, phase separation, and leakage under mechanical loading [16,18,19]. In parallel, LM systems display dynamic interfaces governed by oxide-skin formation, wetting transitions, and compositional evolution, all of which strongly influence both mechanical stability and electrochemical performance [12,14,15,20,21,22].

These LM-specific challenges necessitate a fundamental redefinition of the binder’s role. In LM composite electrodes (Figure 2B), the binder functions as an active matrix that confines LM droplets, stabilizes interfaces, and maintains conductive percolation under deformation. Rather than functioning solely as an adhesive, the binder must act as a multifunctional matrix that actively regulates electro-chemo-mechanical behavior. As shown in Figure 2B, LM composite electrodes incorporate dispersed liquid metal droplets within a polymer network, where the binder governs droplet confinement, suppresses coalescence and leakage, and maintains conductive pathways under deformation [16,18,19,29].

As conceptually illustrated in Figure 2C,D, the transition from passive to active binder functionality arises from a fundamental shift in how the binder regulates structural, interfacial, and transport behavior across multiple length scales [23,24,27,46]. In conventional systems, the binder interacts weakly with particle surfaces through nonspecific interactions such as van der Waals forces, providing cohesion without adapting to stress or interfacial evolution [23,24,27,46]. As a result, the conductive network remains externally defined and is prone to irreversible disruption under deformation [24,27].

In contrast, in LM-based systems, the binder engages in dynamic and interfacially active interactions with the composite (Figure 2D). The presence of a native oxide layer on LM droplets provides chemically active sites that enable strong and adaptive binder–LM interactions, enhancing interfacial stability [10,14,15,46]. Simultaneously, the polymer matrix exerts mechanical confinement that counteracts surface-tension-driven coalescence, maintaining a stable droplet dispersion even under strain [8,12,16,17,18]. Viscoelastic and dynamically crosslinked polymer networks further redistribute local stress throughout the matrix, thereby suppressing crack formation and structural failure during repeated deformation [7,15,29,30,47]. Reversible intermolecular interactions allow the binder network to reform after mechanical damage, while the fluidic LM phase restores conductive pathways, leading to self-healing of both structure and electrical functionality [15,29,30]. For example, Zhao et al. demonstrated that noncovalently crosslinked LM-incorporated polymer binders improved stress tolerance and electrochemical stability in Si microparticle anodes through dynamic ion–dipole and hydrogen-bonding interactions [7]. Together, these mechanisms help preserve electrical continuity and structural integrity during deformation and microstructural rearrangement [15,29,30,32,33,52].

Through these mechanisms, the binder evolves from a passive mechanical component into an active regulator of droplet stability, interfacial chemistry, stress dissipation, and transport continuity. This expanded role is supported by recent experimental studies, including noncovalently crosslinked liquid metal-incorporated polymer binders that enhance adhesion and mechanical robustness in silicon-based electrodes, demonstrating the direct influence of binder chemistry on electrochemical performance [7,15,29,30].

It can be concluded that conventional binders are limited to mechanical support and cannot maintain electrical connectivity under stress, whereas in LM-based systems, the binder functions as an integrated regulator that couples mechanical confinement, interfacial stability, and transport behavior. This shift is particularly critical for soft energy storage devices, where performance depends on the interplay of deformation, charge transport, and interfacial evolution [1,5,7]. This evolution from passive cohesion toward multifunctional electro–chemo–mechanical regulation is summarized in Table 1, highlighting the progression from conventional adhesive binders to dynamically active LM-compatible matrices.

Rather than representing a strict binary distinction, binder functionality spans a continuum from passive cohesion to active electro-chemo-mechanical regulation, as illustrated in Table 1 [23,24,27,28,46,47]. Classical binders such as PVDF represent the passive regime, while intermediate systems such as CMC/SBR, poly(acrylic acid), and polysaccharide-based binders introduce stronger interfacial interactions and improved mechanical compliance [24,27,28]. At the advanced end of this spectrum, elastomeric and LM-compatible binders, including styrene–isoprene–styrene (SIS) and dynamic polymer networks, function as active matrices that regulate droplet confinement, interfacial stability, rheological behavior, and damage tolerance [15,29,30]. Recent SIS-based LM composites and reconfigurable 3R battery architectures, where R stands for resilience, repairable and recyclable, further demonstrate that binder design can simultaneously regulate rheology, mechanical compliance, interfacial adhesion, and recyclability in multifunctional soft energy devices [48,49,50,52]. This progression highlights a fundamental shift in binder design philosophy toward multifunctional, adaptive materials for resilient LM-based energy storage systems.

## 3. Fundamental Interactions Between Binders and LMs

The behavior of LM composites in soft energy storage systems is governed by coupled interactions spanning droplet-scale physics, oxide-mediated interfacial chemistry, and polymer matrix mechanics. Prior studies have examined LM dispersion and printability in composite inks [18], dynamic interfacial behavior in LM electrochemical systems [15], and the role of functional polymer binders in battery electrodes [46,47]. In addition, recent studies on biphasic elastomer–LM composites and printable LM architectures demonstrated that polymer matrices critically influence LM dispersion stability, rheological behavior, and conductive network formation during processing [48,49,50,51]. However, these aspects are typically treated independently, without considering their intrinsic coupling.

**Figure 3:**
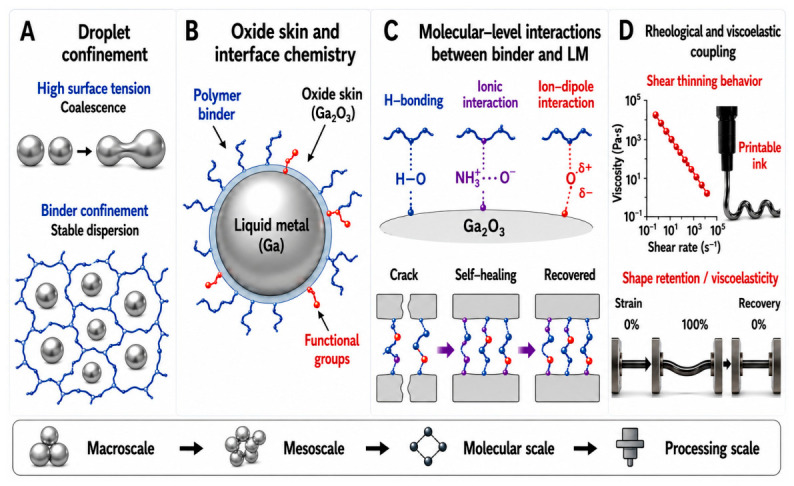
Multiscale binder–liquid metal interactions in composite systems. (**A**) Binder-mediated confinement suppresses droplet coalescence and stabilizes liquid metal dispersion. (**B**) The native oxide skin enables interfacial interactions between LM and polymer binders through surface functional groups. (**C**) Dynamic molecular interactions, including hydrogen bonding, ionic interactions, and ion–dipole interactions, contribute to adhesion, self-healing, and structural adaptability. (**D**) Rheological and viscoelastic properties govern processability, printability, and shape retention. Together, these binder-regulated interactions operate across multiple length scales, from droplet stabilization to molecular regulation and manufacturing performance in soft energy storage systems.

In contrast to conventional composite electrodes dominated by rigid solid–solid contacts, LM composites incorporate a fluid metallic phase within a deformable polymer matrix, resulting in strong coupling between surface-tension-driven droplet behavior, oxide-controlled interfacial interactions, and binder-governed viscoelastic response. This coupling fundamentally alters the mechanisms governing structural stability and electrochemical performance. As summarized in Figure 3, these coupled mechanisms span multiple length scales, linking droplet-scale confinement, oxide-mediated interfacial chemistry, molecular-level binder interactions, and macroscopic rheological behavior. At the microscale, surface tension (~500–700 mN/m depending on alloy composition and oxidation state) drives droplet spheroidization, coalescence, and redistribution, directly influencing conductive percolation and microstructural stability [18,64,65]. At the interfacial level, the native oxide layer on LM droplets mediates dynamic chemical interactions with polymer functional groups and electrolytes, governing wetting behavior, adhesion, and charge transfer processes [15]. At the mesoscale, the polymer binder provides viscoelastic confinement and mechanical adaptability, enabling stress redistribution and maintaining structural integrity under deformation [46,47].

### 3.1. Droplet Physics and Confinement in Polymer Matrices

A defining physical feature of Ga-based and related room-temperature LMs is their high surface tension, which drives droplet spheroidization and hinders controlled dispersion within composite matrices [18]. In printable LM nanocomposites, this behavior is recognized as a key processing challenge, as it promotes droplet aggregation, reduces microstructural uniformity, and limits pattern fidelity in the absence of effective stabilization strategies [18]. As illustrated in Figure 3A, surface tension-driven coalescence leads to unstable droplet distributions when confinement is insufficient, disrupting conductive percolation and structural homogeneity.

In this context, the binder plays a fundamental role by providing physical confinement and kinetic stabilization of the LM phase, enabling uniform droplet dispersion within the polymer matrix. This confinement introduces a balance between capillary forces and matrix elasticity, which suppresses coalescence and stabilizes droplet distribution under both static and dynamic conditions. Recent studies on elastomer-confined EGaIn composites further showed that polymer network elasticity can kinetically stabilize LM droplets and suppress deformation-induced coalescence during repeated mechanical loading [48,49,50,51].

This function becomes particularly critical under repeated mechanical deformation, where droplet rearrangement, migration, and coalescence can otherwise accelerate. Although the intrinsic fluidity of LM phases enables adaptive deformation and conductivity retention, this advantage is only realized when the surrounding polymer matrix effectively constrains droplet motion [12]. Without such confinement, deformation-induced redistribution can lead to loss of percolation pathways and electrical discontinuity. Therefore, binder-mediated confinement is not merely a structural feature but a key determinant of conductive network stability in LM composites. By regulating droplet spacing, limiting coalescence, and preserving percolation pathways, the binder directly governs the ability of LM-based systems to maintain electrical continuity under mechanical strain.

### 3.2. Oxide Skin and Binder–LM Interfacial Chemistry

Ga-based LMs rapidly form a thin native oxide skin under ambient conditions, which fundamentally governs their interfacial behavior. This oxide layer strongly influences wetting, adhesion, and surface mechanics, distinguishing LM interfaces from those of conventional liquids or rigid metals [66,67,68]. As illustrated in Figure 3B, the polymer binder interacts primarily with the oxide-containing surface layer, which may include Ga_2_O_3_, Ga_2_O, oxyhydroxide species, and hydroxylated Ga oxide surface groups rather than with the metallic core, resulting in an interface that is chemically active and mechanically adaptive.

The oxide layer serves as a critical interfacial mediator by providing reactive sites for interaction with polymer functional groups, including hydroxyl, carboxyl, and ionic moieties. These interactions enhance adhesion between LM droplets and the surrounding polymer matrix while stabilizing the droplet–matrix interface under deformation [67,68,69,70]. For instance, oxide-mediated interactions between Ga-based LM droplets and polar polymer matrices have been shown to significantly improve wetting behavior, interfacial adhesion, and structural stability in stretchable composite systems [67,70]. Unlike conventional solid–solid contacts, where interfaces are largely static, LM interfaces are dynamic and responsive, enabling continuous reconfiguration under mechanical or electrochemical perturbations.

In electrochemical LM systems, interfacial behavior is further complicated by dynamic surface and compositional evolution during operation. Processes such as oxide growth, rupture, and regeneration, along with wetting transitions and ion-mediated interactions, continuously modify interfacial properties and influence charge transfer and stability [71,72]. As a result, the LM interface cannot be treated as a passive boundary but must be considered an active and evolving component of the system. Within this framework, binder chemistry plays a central role in regulating oxide-mediated interactions. By tailoring functional groups and interaction strength, the binder can tune interfacial adhesion, stabilize droplet dispersion, and influence electrochemical processes at the LM interface [73,74,75]. This perspective shifts the role of the binder from passive structural support toward active regulation of LM interfacial behavior.

### 3.3. Molecular-Level Interactions Between Binder and LM

At the molecular level, binder–LM interactions arise from a combination of physical confinement and reversible interfacial interactions. In advanced binder systems, functionality increasingly originates from programmable intermolecular interactions rather than passive cohesion alone, including hydrogen bonding, ionic interactions, metal–ligand coordination, and supramolecular association [76,77]. As illustrated in Figure 3C, polymer functional groups can interact with the oxide-coated LM surface through hydrogen bonding, ionic interactions, and ion–dipole coupling, forming dynamic and physically crosslinked networks [7,74].

These reversible interactions play a critical role in stabilizing the LM–polymer interface by enhancing adhesion, maintaining dispersion, and enabling structural adaptability. Unlike permanent covalent crosslinks, dynamic noncovalent interactions can continuously break and reform under mechanical or electrochemical perturbations, allowing the composite to accommodate deformation while preserving interfacial integrity [61,62,76]. At the same time, these molecular interactions contribute to energy dissipation and stress relaxation within the polymer network. By distributing local stresses and preventing their accumulation at droplet interfaces, they reduce the likelihood of interfacial failure and structural degradation. This behavior is particularly important in LM composites, where repeated deformation and interfacial evolution can otherwise destabilize the system [78].

The effectiveness of this approach is supported by recent studies on noncovalently crosslinked liquid metal-incorporated polymer binders, where multiple dynamic interactions were employed to enhance adhesion, mechanical robustness, and electrochemical stability in silicon-based electrodes [7]. Related self-healing and conductive binder systems based on hydrogen bonding further demonstrate that reversible intermolecular interactions can be strategically designed to regulate mechanical recovery, interfacial contact, and charge transport continuity in deformable electrochemical systems [58,60]. As a result, molecular-scale binder interactions strongly influence both mechanical adaptability and transport stability in LM composites.

### 3.4. Rheological and Viscoelastic Coupling: From Formulation to Function

Binder–LM interactions propagate from the molecular and interfacial levels to the macroscopic rheological and mechanical response of the composite. In LM-based printable systems, polymer-assisted rheology is essential for transforming a high-surface-tension LM into a processable material that exhibits controlled flow during deposition and shape retention after printing. As illustrated in Figure 3D, binder design governs shear-thinning behavior under applied stress and elastic recovery upon stress release, enabling both printability and structural fidelity [18,79,80,81]. This rheological behavior originates from the dynamic interactions between the polymer network and the dispersed LM droplets. Under shear, reversible interactions and network rearrangement allow flow, while upon relaxation, the reformation of physical crosslinks restores structural integrity. Similar shear-thinning and recovery behavior is widely recognized as essential for direct ink writing, where inks must flow through the nozzle but rapidly recover viscosity or modulus after deposition to retain printed geometry [80,81]. Recent LM printable inks based on dynamically crosslinked elastomeric matrices further demonstrated that optimized viscoelasticity enables stable extrusion, high printing fidelity, and mechanically robust conductive architectures without post-sintering processes [76,77,78].

Importantly, rheological control extends beyond fabrication and directly impacts device performance. In soft energy systems, LM composites must withstand repeated mechanical deformation while preserving conductive pathways. The binder therefore defines a coupled viscoelastic response: it must be sufficiently compliant to accommodate strain while maintaining enough cohesion to suppress droplet rearrangement, leakage, and disruption of conductive networks. This balance determines whether the composite can retain electrical continuity under dynamic conditions [50,63,82]. Taken together, the behavior of LM composites is governed by the coupling of surface-tension-driven droplet physics, oxide-mediated interfacial chemistry, reversible binder–LM interactions, and binder-controlled rheology [18]. As summarized in Figure 3, these mechanisms operate across multiple length scales, from droplet confinement and interfacial stabilization to macroscopic viscoelastic response. Together, these coupled mechanisms explain how binder design influences droplet stability, rheology, and electrochemical reliability in soft LM-based energy systems.

## 4. Binder Engineering for Structural Stability and Conductive Percolation

The performance of LM composites in soft energy storage critically depends on the ability to simultaneously preserve structural integrity and maintain electrically continuous pathways during deformation and electrochemical cycling. This requirement is more stringent than in conventional particulate electrodes because LM-containing systems are intrinsically dynamic: the metallic phase can deform, redistribute, and coalesce if not effectively constrained. Consequently, binder design emerges not merely as a means of adhesion, but as a primary determinant of structural stability and functional reliability under operating conditions [83,84,85].

**Figure 4:**
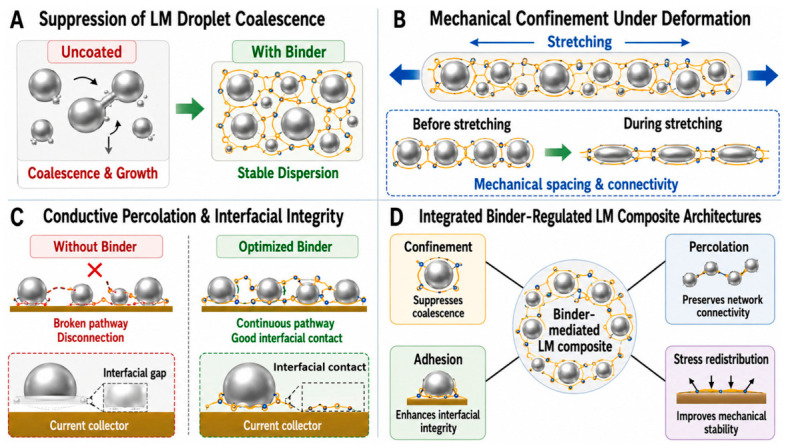
Binder engineering for structural stability and conductive percolation in liquid metal composites. (**A**) In the absence of binder confinement, LM droplets undergo surface-tension-driven coalescence and growth, whereas binder-mediated confinement stabilizes droplet dispersion. (**B**) The polymer matrix maintains droplet spacing and network connectivity during mechanical deformation, preserving conductive pathways under strain. (**C**) Weak binder systems lead to interfacial gaps and disrupted conductive pathways, while optimized binders promote conformal contact, strong interfacial adhesion, and continuous percolation. (**D**) Schematic illustration of a binder-regulated LM composite architecture, highlighting the coupled roles of confinement, conductive percolation, interfacial adhesion, and stress redistribution in maintaining structural integrity and electrical continuity.

A central challenge is the suppression of droplet coalescence. Ga-based and related LMs exhibit high surface tension, which favors minimization of interfacial area and promotes droplet spheroidization and merger. In printable LM composites, this behavior is widely recognized as a major obstacle to stable processing, uniform microstructure, and pattern fidelity, necessitating strategies for stabilizing LM dispersions [18,50,86]. Within such systems, the binder provides mechanical confinement and resistance to droplet migration, thereby suppressing surface tension-driven coalescence and stabilizing droplet distributions. Recent biphasic LM–elastomer systems based on SIS matrices demonstrated that elastomeric confinement can effectively suppress LM droplet aggregation and preserve dispersion uniformity during repeated deformation and processing [48,49,50,51]. In addition to polymer-mediated confinement, stabilization of LM phases has also been explored through interfacial adhesion between LM droplets and functional particle surfaces, including carbon-, graphene-, and metal-coated fillers, which can further suppress droplet migration and leakage during deformation and processing [87,88]. These systems include conductive Ag-coated LM composites, printable biphasic LM elastomers, and recyclable 3R electronic architectures that rely on polymer-mediated stabilization of the LM phase [48,49,50,51]. This function becomes particularly important under repeated deformation, where droplet rearrangement and merger would otherwise accelerate. As illustrated in Figure 4A, binder-mediated confinement maintains uniform LM dispersion and mitigates coalescence within the composite matrix.

Beyond droplet stabilization, the binder must preserve conductive percolation. In LM-based conductors, electrical continuity can be retained under strain due to the fluidic adaptability of the metallic phase [12,85,89]. However, this intrinsic advantage is only effective when the surrounding matrix maintains spatial connectivity between LM domains and prevents excessive separation or phase redistribution. In this context, the binder helps maintain inter-droplet connectivity by regulating droplet spacing, matrix elasticity, and interfacial compatibility. As shown in Figure 4B, binder-mediated confinement preserves inter-droplet connectivity under strain, enabling stable conductive pathways during deformation [83,90]. For example, stretchable LM composite conductors and Ga–CB–SIS battery electrodes demonstrated that optimized polymer matrices can preserve inter-droplet contact and conductive pathways even under large tensile strain and cyclic deformation [43,48,89]. Thus, electrical stability arises from the coupled behavior of the LM phase and the binder, rather than from the LM phase alone.

Binder-mediated adhesion and interfacial integrity are equally critical. In conventional composite electrodes, adhesion to the current collector and cohesion within the electrode are key determinants of mechanical stability and electrochemical durability. For example, in silicon-based electrodes, the balance between adhesion and cohesion directly influences electrode integrity and cycling performance [91,92,93]. In LM composites, this challenge is amplified by the presence of a fluid metallic phase and continuously evolving internal interfaces. The binder must therefore establish robust coupling not only between active materials and the substrate but also between LM droplets and the surrounding matrix, preventing delamination, structural collapse, and electrical disconnection under deformation. In multilayer LM-based soft electronic architectures, elastomeric binder systems have been shown to improve conformal interfacial contact and suppress delamination between conductive layers and deformable substrates during repeated mechanical loading [49,51]. As illustrated in Figure 4C, weak binder systems lead to disrupted conductive pathways and interfacial gaps, whereas optimized binders maintain continuous percolation and strong interfacial adhesion.

These design requirements are supported by recent advances in LM-containing binder and composite systems. Noncovalently crosslinked liquid metal-incorporated polymer binders have been shown to enhance adhesion, mechanical robustness, and electrochemical stability in silicon microparticle anodes, demonstrating that binder chemistry can actively regulate structural integrity rather than merely provide passive cohesion [7]. Related LM–elastomer and self-healing LM composites further demonstrate that matrix design can enable reconfigurable conductive networks, electrical recovery after damage, and stable electromechanical behavior under deformation [63,94,95]. This finding is particularly relevant for soft energy storage systems, where repeated deformation and structural evolution demand adaptive and resilient material architectures.

Overall, structural stability and conductive pathway retention in LM composites are strongly governed by binder design. Effective binders do not simply immobilize the metallic phase; they stabilize droplet distributions, maintain electrical connectivity, reinforce interfaces, and help the composite accommodate deformation without catastrophic loss of function. As summarized in Figure 4D, these coupled functions are essential for the long-term mechanical and electrochemical reliability of soft LM-based energy systems [44,50,63].

## 5. Dynamic and Self-Healing Binder Systems

**Figure 5:**
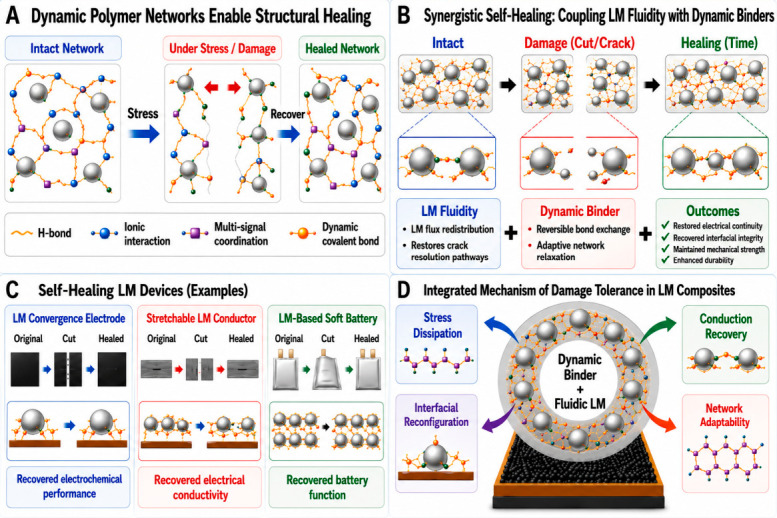
Dynamic and self-healing behavior in LM composite systems. (**A**) Reversible interactions within dynamic polymer networks enable structural healing after mechanical damage. (**B**) Coupling of LM fluidity and dynamic binders promotes conductive pathway reconstruction and network recovery during self-healing. (**C**) Representative self-healing LM composite devices, including electrodes, stretchable conductors, and soft batteries. (**D**) Schematic illustration of damage tolerance in LM composites, highlighting the cooperative roles of dynamic binders and fluidic LM in stress dissipation, interfacial reconfiguration, conductive recovery, and structural adaptability.

The realization of reliable soft energy storage systems based on LM composites requires materials capable of accommodating simultaneous mechanical deformation, interfacial evolution, and electrochemical cycling. Under such conditions, conventional static binders fail due to the accumulation of irreversible damage, including crack formation, interfacial separation, and conductive network disruption. Dynamic and self-healing binder systems address these limitations by introducing reversible interactions that enable continuous structural reconfiguration and functional recovery. As summarized in Figure 5, self-healing in LM composites emerges from the multiscale coupling between dynamic polymer networks and fluidic LM domains, linking molecular-scale reversible interactions with macroscopic conductive recovery [60,61,76].

### 5.1. Dynamic Bonding and Reversible Polymer Networks

Dynamic binder systems derive their functionality from reversible intermolecular interactions, including hydrogen bonding, ionic interactions, metal–ligand coordination, supramolecular association, and dynamic covalent bonds. These interactions create adaptable polymer networks capable of stress-induced bond dissociation followed by spontaneous reformation, enabling structural relaxation and recovery [62,76,77]. For example, Zhao et al. developed a LM-incorporated polymer binder containing dynamic ion–dipole interactions and hydrogen-bonding networks that enabled enhanced stress dissipation and improved cycling stability in silicon microparticle anodes [7]. Similarly, self-healing elastomeric systems based on reversible supramolecular interactions have demonstrated rapid mechanical recovery and conductivity restoration after repeated deformation [76,78]. In advanced battery systems, such binders have been shown to enhance electrode integrity and cycling stability, reflecting a transition from passive adhesion to active mechanical regulation [46,47]. In LM composites, this concept is further realized through elastomeric matrices such as styrenic block copolymers (e.g., SIS), which form physically crosslinked networks via microphase separation. As illustrated in Figure 5A, these non-permanent junctions provide a dynamic framework that accommodates large deformation while preserving structural cohesion. Under external stimuli, increased chain mobility enables network reconfiguration and reconnection at damaged interfaces. In LM-based stretchable battery systems, SIS-based binders provide hyperelastic support and reversible structural reorganization, sustaining both mechanical integrity and electrochemical performance under repeated deformation [43,44,63]. Related dynamic elastomeric systems have also shown recovery of conductivity and mechanical resilience after repeated mechanical damage [78,94].

### 5.2. Synergistic Self-Healing: Coupling LM Fluidity with Dynamic Binders

A defining characteristic of LM composites is that self-healing emerges from the coupling of polymer dynamics with LM fluidity. Dynamic binders enable structural recovery through reversible bonding, while the LM phase contributes through its intrinsic ability to flow, reconfigure, and re-establish conductive pathways. For instance, stretchable LM composite conductors reported by Lee et al. exhibited autonomous restoration of electrical conductivity after mechanical rupture due to fluidic LM redistribution within a deformable polymer matrix [89]. Related LM–elastomer composites further showed that reversible polymer confinement can guide conductive network reconstruction during repeated mechanical cycling [94]. As illustrated in Figure 5B, mechanical damage disrupts both structural continuity and electrical connectivity, while healing involves simultaneous polymer network reformation and LM redistribution.

Ga-based LM systems exhibit intrinsic self-healing electrical behavior because the liquid phase can autonomously restore conductive continuity following mechanical or electrochemical disruption [89,94]. This response contrasts sharply with rigid conductive fillers, which typically undergo irreversible fracture and permanent loss of percolation. At the composite level, binder design amplifies this advantage by constraining LM redistribution while enabling network reconnection, thereby ensuring recovery of both mechanical integrity and electrical conductivity [7,95]. As a result, self-healing behavior in LM composites depends on the cooperative interaction between reversible polymer networks and the fluidic LM phase.

### 5.3. Self-Healing Electrochemical Interfaces

Dynamic binder systems also regulate electrochemical interfaces, which are inherently unstable in LM-based systems due to oxide formation, wetting transitions, interfacial rupture, and compositional evolution. These processes can disrupt charge transport and degrade performance if interfacial contact is not continuously maintained [15,72].

Adaptive binder networks mitigate these effects by preserving and restoring interfacial contact during structural evolution. Recent LM-enabled battery systems demonstrated that dynamic polymer matrices can maintain conformal LM–electrode contact during repeated deformation and electrochemical cycling, thereby reducing interfacial degradation and preserving transport continuity [20,43,44,63]. In conventional composite electrodes, binder-mediated adhesion and cohesion strongly influence interfacial stability and electrochemical durability [91,92]. In LM systems, this role becomes even more critical due to the presence of fluid phases and continuously evolving interfaces. The combination of dynamic polymer networks and fluidic LM domains enables interfaces to reconfigure rather than degrade irreversibly, restoring both ionic and electronic pathways after damage [7,43,63]. Such adaptive interfacial behavior is important for maintaining electrochemical stability during repeated deformation and cycling.

### 5.4. Toward Damage-Tolerant Soft Energy Systems

The integration of dynamic binders with LM phases enables a transition from damage avoidance to damage tolerance and functional resilience. In LM-based systems, devices have been demonstrated to remain operational after mechanical damage or to recover functionality under external stimuli, demonstrating device-level self-healing behavior. As shown experimentally in Figure 5C, Ga–CB–SIS composite systems can recover structural continuity after severe mechanical disruption, demonstrating restoration of both mechanical integrity and conductive functionality [44,63]. Related reconfigurable LM electronic systems and self-healing conductors have also demonstrated recovery of conductivity after cutting, stretching, or cyclic mechanical damage, highlighting the importance of dynamic polymer confinement in enabling device-level resilience [78,89,94]. Such capabilities are particularly important for wearable and biointegrated electronics, where mechanical damage and repeated deformation are unavoidable. By enabling simultaneous recovery of structure, conductivity, and interfacial integrity, dynamic binder systems provide a pathway toward long-lifetime, mechanically robust, and adaptive soft energy storage technologies [1,94].

Overall, self-healing in LM composites arises from the interaction between reversible polymer networks and fluidic LM phases. Dynamic binders provide adaptable structural support and help regulate interfacial stability, while LM domains restore electrical continuity through autonomous reconfiguration. As summarized in Figure 5, these coupled mechanisms enable LM-based soft energy systems to maintain functionality under repeated mechanical and electrochemical perturbations [43,44,51]. Future progress will depend on designing binder–LM interactions that balance healing efficiency, interfacial stability, viscoelastic adaptability, and electrochemical compatibility.

## 6. Binder-Controlled Electrochemical Interfaces

**Figure 6:**
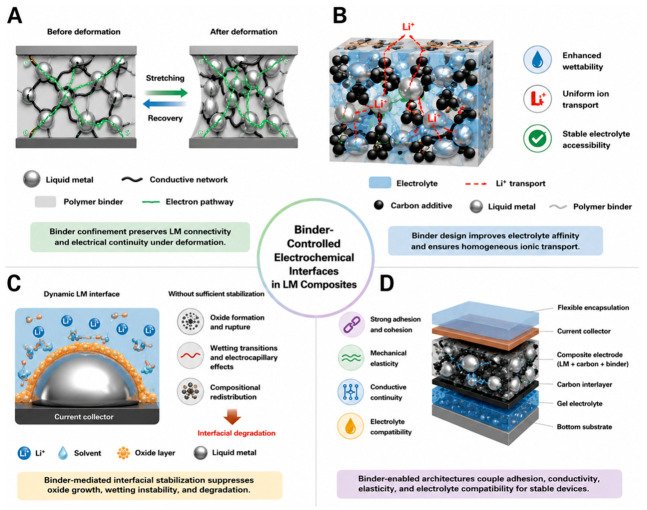
Binder-controlled electrochemical interfaces in LM composites. (**A**) Binder confinement maintains electrical continuity during deformation. (**B**) Binder-regulated electrolyte accessibility enables uniform ionic transport. (**C**) Binder-mediated stabilization suppresses degradation at dynamic LM electrochemical interfaces. (**D**) Integrated LM composite architectures couple adhesion, conductivity, elasticity, and electrolyte compatibility for stable soft energy storage devices.

Electrochemical performance in LM composite electrodes is governed by how the binder regulates electronic connectivity, ionic accessibility, and interfacial stability. Polymer binders are increasingly recognized as active electrochemical components that influence electrode microstructure, charge transport pathways, electrolyte wetting, and electrode–electrolyte interfacial evolution rather than serving solely as passive structural additives [25,26,96,97]. In LM systems, this role is further amplified by the presence of a fluid metallic phase and dynamically evolving interfaces, which intrinsically couple mechanical deformation with electrochemical behavior. As summarized in Figure 6, binder-controlled interfaces operate through coupled interfacial mechanisms that determine whether conductive networks remain connected, ionic transport remains spatially uniform, and LM surfaces remain electrochemically stable during operation.

### 6.1. Maintenance of Electrical Contact

A primary function of the binder is to preserve electronic connectivity within the composite electrode. In conventional composite electrodes, binder-mediated adhesion and cohesion directly influence fracture resistance, contact retention, and cycling stability, particularly in electrodes undergoing repeated volume changes [92,93]. In LM composites, this challenge is compounded by the fluidity of the conductive phase. Although Ga-based LMs can sustain conductivity under large deformation, this behavior remains dependent on matrix-mediated confinement that prevents excessive droplet migration and maintains inter-domain connectivity [85]. For example, recent stretchable and self-healable lithium-ion batteries based on dynamic covalent polymer networks showed that deformable polymer matrices can preserve electrical contact and electrochemical operation under mechanical strain, highlighting the importance of mechanically adaptive interfacial design in soft energy storage systems [98].

Binder-mediated confinement stabilizes inter-droplet spacing and suppresses droplet redistribution, thereby preserving conductive percolation and preventing electrical discontinuity. As shown in Figure 6A, multilayer composite architectures leverage binder design to maintain electrical continuity across interfaces while preventing delamination. In these systems, the binder simultaneously ensures internal cohesion and interfacial coupling between layers, enabling stable current flow under coupled mechanical and electrochemical perturbations [43,44,63].

### 6.2. Regulation of Ionic Transport and Electrolyte Accessibility

Beyond electronic conduction, the binder regulates ionic transport by controlling electrode microstructure, wettability, electrolyte accessibility, and local ion-transport pathways. Molecular-level studies of functional binders demonstrate that polymer architecture can directly influence ion mobility, electrolyte uptake, and charge-transfer homogeneity in composite electrodes [96,97]. Polar and ionically interactive binder systems can improve electrolyte affinity and maintain structural uniformity during cycling, thereby reducing transport heterogeneity and interfacial degradation [25,26]. Similarly, supramolecular ion-conducting polymers have been used as stretchable binders to decouple mechanical toughness from ionic conductivity, enabling highly deformable lithium-ion battery electrodes with efficient ion transport [99]. This role becomes especially important in LM composites, where the fluid metallic phase can dynamically rearrange and continuously alter local transport pathways. As illustrated in Figure 6B, Ga–CB–SIS composite anodes maintain structural integrity during electrolyte exposure and cycling, indicating that the polymer–carbon matrix stabilizes both LM distribution and electrolyte-accessible regions [63]. By suppressing phase segregation and maintaining microstructural homogeneity, binder-mediated confinement enables more spatially uniform ionic transport and minimizes localized transport bottlenecks. Beyond transport regulation, binder chemistry can influence electrochemical interfacial processes during cycling. Polymer–electrolyte interactions affect electrolyte wetting and ion accessibility within LM composite electrodes, thereby influencing charge-transfer kinetics. In systems operating with conventional battery electrolytes, binder chemistry may also influence the formation, uniformity, and stability of the solid–electrolyte interphase (SEI), which plays a critical role in regulating parasitic reactions, ion transport, and long-term electrochemical performance. These effects further contribute to improved cycling stability by maintaining more uniform and electrochemically stable electrode–electrolyte interfaces [25,26,96,97].

### 6.3. Stabilization of Dynamic Electrochemical Interfaces

Electrochemical interfaces in LM systems are inherently dynamic, undergoing continuous evolution through oxide formation, electrocapillary effects, wetting transitions, corrosion, and compositional redistribution. In LM electrochemical systems, these interfacial processes directly govern charge transfer behavior, electrochemical stability, and long-term device performance [72,100,101]. Surface oxidation and electrochemically induced interfacial tension changes can further modify LM morphology and interfacial activity during operation. Recent electrochemical studies of EGaIn further showed that oxide formation and removal can strongly modulate interfacial tension and surface activity, directly linking electrochemical state to LM morphology and interfacial stability [102].

Binder design provides a means to stabilize these dynamic interfaces. By maintaining mechanical confinement, electrolyte compatibility, and interfacial continuity, the binder ensures that LM surfaces remain electrochemically accessible while suppressing uncontrolled degradation. This concept is schematically illustrated in Figure 6C, where surface chemical evolution of a liquid metal-coated electrode reveals heterogeneous oxidation and compositional redistribution in the absence of sufficient interfacial stabilization. Recent studies on Ga-based LM corrosion further demonstrate that electrolyte-induced interfacial reactions can destabilize LM phases if not regulated by the surrounding matrix [103]. These observations indicate that electrochemical stability in LM systems depends strongly on matrix-controlled interfacial regulation rather than on the metallic phase alone.

### 6.4. Interfacial Design in LM Composite Architectures

These interfacial functions are realized through binder-enabled material and device architectures. In stretchable Ga-based batteries, Ga–Carbon–SIS composites integrate conductivity, adhesion, printability, and mechanical resilience within a single matrix, enabling stable operation under deformation and electrolyte exposure [43,63]. Similarly, in 3R battery architectures, SIS-based composites are employed across multiple printed layers to provide cohesion, interfacial adhesion, elasticity, and repairability, while carbon-based interlayers mitigate electrolyte-induced degradation of conductive components [44,51]. At lower carbon-to-Ga ratios, Ga droplet leakage and aggregation occur, directly demonstrating that interfacial stability and transport uniformity depend critically on binder-mediated confinement [63]. These examples establish a general design principle: interfacial stability in LM systems is not an intrinsic property of the metallic phase but rather a consequence of binder-controlled composite architecture that couples confinement, interfacial compatibility, conductive percolation, and electrolyte-accessible transport pathways. Liquid metal-based stretchable Zn-ion batteries for electronic textiles further demonstrate that stable device-level electrochemical performance requires simultaneous regulation of deformable current collection, interfacial contact, and mechanically compatible device architecture [104]. Overall, binder design strongly influences electrical contact, ionic transport, and interfacial stability in LM composite electrodes. As summarized in Figure 6D, the binder helps maintain conductive pathways, stabilize electrolyte-accessible interfaces, and regulate dynamically evolving LM surfaces during cycling. These functions are essential for achieving mechanically robust and electrochemically stable soft LM-based energy systems.

## 7. Binder-Enabled Processability and Printability

**Figure 7:**
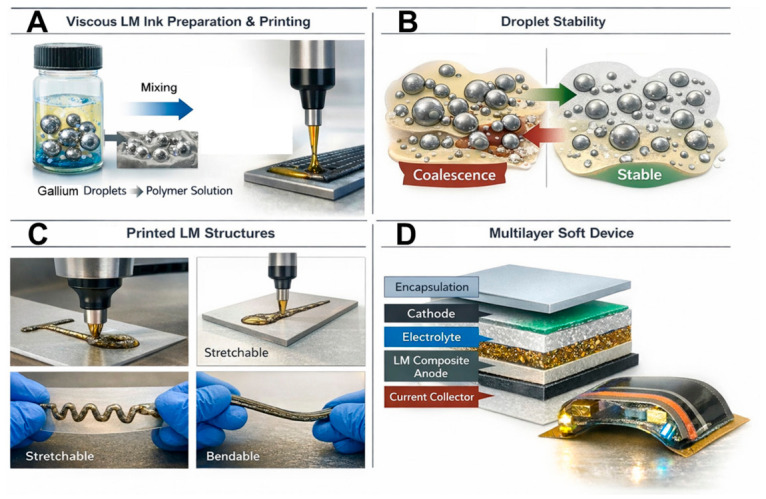
Binder-enabled processability and printability of LM composites. (**A**) Formation of viscoelastic LM inks through polymer-assisted dispersion and shear-thinning behavior. (**B**) Binder-mediated stabilization suppresses LM droplet coalescence and maintains uniform dispersion during processing. (**C**) Extrusion-based printing of stretchable and bendable LM composite structures with preserved mechanical compliance. (**D**) Multilayer soft-device architectures enabled by binder-controlled adhesion, rheology, and interfacial compatibility.

A major advantage of LM composites for soft energy storage lies in their compatibility with room-temperature, additive, and substrate-tolerant fabrication. However, this processability does not originate from the LM itself. Ga-based LMs, despite their metallic conductivity and deformability, exhibit high surface tension, low viscosity, and poor pattern fidelity, leading to uncontrolled spreading and limiting direct printability. Consequently, printable LM systems rely on matrix engineering to transform fluid metals into deposition-compatible composites. Experimental and review studies consistently demonstrate that polymer binders govern processability by regulating rheology, stabilizing dispersed droplets, and enabling adhesion to substrates [18,50,79]. As illustrated in Figure 7, binder-enabled printability emerges from the coupled regulation of rheological behavior, phase stability, structural formation, and device-level integration. In addition to direct ink writing and extrusion-based approaches, LM deposition has also been demonstrated through spray coating, jetting, and piezoelectric jetting techniques. Furthermore, LM printability can be modified through the incorporation of particles, oils, or other additives that influence viscosity, droplet stability, and substrate wetting behavior [105,106].

### 7.1. Rheological Control and Ink Formation

At the ink level, binder design determines whether LM dispersions exhibit the non-Newtonian behavior required for printing. Polymer incorporation introduces viscoelasticity, enabling shear-thinning behavior in which viscosity decreases under applied shear and recovers after deposition. Rheological studies of LM composite systems show that this transition from a Newtonian liquid to a viscoelastic ink is essential for controlled extrusion and post-print shape retention [79]. For example, Neumann et al. demonstrated that polymer-thickened LM inks exhibited pronounced shear-thinning behavior and rapid viscoelastic recovery, enabling stable direct-ink-writing of stretchable conductive structures under ambient conditions [80]. Specifically, LM–polymer inks maintain high viscosity at low shear rates to prevent structural collapse after deposition, while viscosity decreases under high shear during printing to enable continuous nozzle flow. As illustrated in Figure 7A, binder-regulated rheology transforms dispersed LM droplets into stable printable inks, demonstrating that processability arises from matrix-controlled viscoelasticity rather than intrinsic LM properties.

### 7.2. Droplet Stabilization and Phase Control

Binder design also governs the stability of LM dispersions during processing. Due to their high surface tension, LM droplets tend to coalesce, resulting in phase separation and loss of print fidelity. Experimental studies directly demonstrate this behavior: in Ga–CB–SIS composites, reducing the carbon or binder content leads to droplet leakage and aggregation, producing nonuniform structures and failed printability [79]. These observations confirm that stable LM inks require sufficient matrix confinement to counteract surface-tension-driven coalescence. Similarly, functionalized LM dispersions stabilized through polymer-assisted interfacial interactions were shown to suppress droplet aggregation and improve printing fidelity in soft conductive architectures [107]. As shown in Figure 7B, binder-mediated confinement maintains uniform droplet dispersion and suppresses droplet merging during extrusion and deposition. Similar stabilization mechanisms have been reported in biphasic LM composites and functionalized LM inks, where polymer matrices or surface modifications suppress coalescence and enable sinter-free printing of conductive structures [50,107]. These studies indicate that phase stability in LM inks depends strongly on matrix-regulated droplet confinement and interfacial stabilization. However, leakage control and phase stability are also influenced by additional factors, including LM viscosity, droplet size, filler characteristics, particle–LM interactions, and the nature of interparticle bonding. Therefore, effective stabilization generally requires a holistic design approach that considers both binder properties and composite microstructure.

### 7.3. Structural Formation and Mechanical Compliance

Binder-enabled LM inks can be directly printed into mechanically compliant architectures that retain electrical functionality under deformation. Experimental studies show that LM–polymer composites preserve conductivity during stretching and bending because the polymer matrix accommodates deformation while LM domains reconfigure without fracture [81]. This behavior contrasts sharply with rigid conductive inks, where cracking causes irreversible electrical failure. Won et al. further demonstrated that LM–elastomer composite conductors retained stable conductivity under repeated tensile deformation due to the synergistic interaction between deformable polymer matrices and reconfigurable LM pathways [81]. As illustrated in Figure 7C, extrusion-based printing produces stretchable structures that maintain conductive continuity under strain, highlighting the synergistic coupling between LM fluidity and polymer elasticity. Studies on LM-embedded elastomers further demonstrate that ink formulations can be tailored for three-dimensional printing of soft functional materials, where the polymer matrix simultaneously governs flow behavior through viscoelastic regulation, mechanical compliance through elastomeric deformation, and structural stability by maintaining droplet confinement and suppressing phase separation during and after printing [79,81].

### 7.4. Multilayer Integration and Device Fabrication

Binder-controlled processability is essential for integrating LM composites into functional soft energy devices. In printed and deformable systems, multiple functional layers—including electrodes, current collectors, electrolyte interfaces, and encapsulation layers—must be assembled without high-temperature processing while maintaining electrical continuity, interfacial adhesion, and mechanical compliance. Unlike conventional metallic electrodes that often require sintering or rigid assembly, LM composites rely on polymer-assisted interfacial compatibility to achieve cohesive multilayer architectures under room-temperature fabrication conditions [79,107].

Recent studies on printable LM systems demonstrate that polymer matrices are central to enabling additive manufacturing of multilayer soft electronics. Hajalilou et al. showed that biphasic LM composites stabilized by polymeric matrices enabled sinter-free fabrication of stretchable conductive structures with robust interfacial integrity during deformation [50]. Similarly, functionalized LM inks with controlled rheological behavior have been used to fabricate multilayer printed conductors and soft electronic architectures while maintaining pattern fidelity and mechanical compliance [107].

In LM-embedded elastomer systems, polymer matrices not only regulate printability but also maintain adhesion between deposited layers and suppress interfacial delamination under repeated strain. Extrusion-printed LM elastomer composites have demonstrated stable conductive performance during bending and stretching due to binder-mediated viscoelastic compatibility between adjacent layers [81]. Related studies on printable LM nanocomposites further showed that matrix-controlled rheology and interfacial stabilization are essential for maintaining structural integrity during multilayer deposition and device fabrication [18]. Related additive-manufacturing studies also demonstrated that elastomer-supported LM architectures could be patterned into multilayer deformable devices while preserving interfacial integrity and mechanical compliance during cyclic deformation [108].

Beyond conductive architectures, binder-controlled multilayer integration is also important for soft energy systems. In 3R battery architectures, SIS-based composites enabled room-temperature fabrication of multilayer deformable batteries without sintering, while maintaining strong interlayer adhesion and structural cohesion during operation [43,44]. Similarly, recyclable LM composite systems demonstrated that elastomeric matrices can maintain pattern stability and interfacial durability in reconfigurable soft electronic devices [94]. These studies collectively highlight that successful LM device fabrication depends not only on LM conductivity but also on binder-mediated regulation of adhesion, rheology, and interfacial mechanics.

As illustrated in Figure 7D, binder engineering enables multilayer integration by simultaneously providing rheological tunability, structural cohesion, interfacial adhesion, and mechanical compatibility across printed layers. In this framework, the binder functions not merely as a passive additive but as a key component linking material formulation with device fabrication and architecture. Overall, printable LM composites rely on the coupled regulation of flow behavior, droplet stability, interfacial compatibility, and mechanical compliance. By suppressing coalescence, enabling compliant conductive architectures, and maintaining stable interlayer adhesion, polymer binders transform intrinsically difficult-to-print LMs into scalable soft electronic and energy storage systems [108,109].

## 8. Applications in Soft Energy Storage

Liquid metal (LM) composites have enabled a new generation of soft energy storage systems, including stretchable batteries, flexible supercapacitors, and multifunctional integrated devices. Across these applications, the primary challenge is not only achieving electrochemical performance but maintaining it under repeated deformation, interfacial evolution, and dynamic operating conditions. This requirement fundamentally depends on binder-mediated stabilization of LM phases, conductive pathways, and electrode interfaces. As a result, application-level functionality in LM systems reflects the effectiveness of binder-controlled coupling between mechanical adaptability and electrochemical behavior.

### 8.1. Stretchable Batteries: LM-Enabled Deformable Electrochemistry

**Figure 8:**
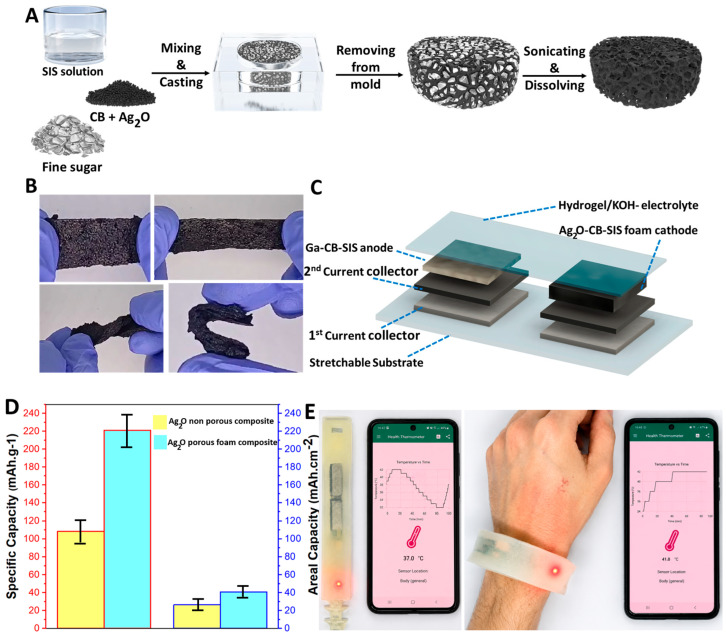
Application of binder-engineered liquid metal composites in soft energy storage systems. (**A**) Fabrication of Ag_2_O–CB–SIS composite foam. (**B**) Mechanical deformability of the foam under stretching, twisting, and bending. (**C**) Schematic configuration of the Ag_2_O–Ga battery. (**D**) Comparison of specific and areal capacities between porous composite foam and nonporous counterparts. (**E**) Demonstration of temperature monitoring as an integrated device application. These examples highlight the role of binder-enabled composite design in achieving mechanical adaptability, electrochemical functionality, and device integration. Reprinted with permission from Ref. [43]. Wiley, 2025.

Stretchable batteries represent one of the most actively explored application areas for LM composites. Unlike conventional rigid electrodes, LM-based electrodes can maintain electrical continuity under large strain because the metallic phase deforms fluidically rather than fracturing [110,111]. Ga-based LM systems further benefit from oxide-stabilized interfaces that support conductive stability during stretching and bending [12]. However, this functionality can only be realized when the LM phase is effectively confined within a stabilizing polymer matrix. In stretchable Ga battery systems, Ga–CB–SIS composite anodes demonstrate that polymer–carbon networks restrict LM redistribution while preserving conductive percolation under deformation [43]. As illustrated in Figure 8B, the resulting porous composite architectures maintain structural integrity under bending, twisting, and tensile deformation. Related studies on LM elastomer electrodes similarly show that inadequate matrix confinement leads to droplet migration, electrical discontinuity, and structural degradation during cycling [83,112]. These results demonstrate that electrochemical stability in stretchable LM batteries depends strongly on polymer confinement and interfacial regulation in addition to the intrinsic conductivity of the LM phase. Additional evidence comes from deformable Zn–LM and hybrid LM battery systems, where polymer-assisted LM stabilization enables mechanically resilient electrochemical architectures capable of operating under repeated strain [113,114]. Such systems demonstrate that binder design directly influences the balance between electrochemical functionality and mechanical deformability. Representative Ag_2_O–Ga soft battery architectures enabled through binder-engineered LM composites are shown in Figure 8C.

### 8.2. Flexible Supercapacitors: Interfacial and Transport Limitations

LM composites have also been explored in flexible and stretchable supercapacitors, where they function as conductive scaffolds, deformable current collectors, or hybrid charge storage electrodes. In these systems, electrochemical performance is dominated by interfacial charge storage and ion accessibility, making surface stability and electrolyte interaction particularly important [115]. Compared with battery systems, LM-based supercapacitors remain less developed due to challenges associated with high surface tension and droplet instability, which reduce effective interfacial area and limit charge storage capability. Studies on LM nanocomposites and LM-based conductive hydrogels show that insufficient dispersion and poor interfacial stabilization can lead to reduced accessible surface area and nonuniform reaction environments, directly affecting capacitance and rate performance [115]. Binder and matrix design address these limitations by regulating wettability, pore structure, and electrolyte accessibility. Functional polymer networks can improve ionic transport and stabilize interfacial charge storage regions by maintaining structural homogeneity and preventing phase segregation during deformation [115]. In LM-based supercapacitors, such regulation is essential for sustaining stable ion-accessible interfaces under repeated mechanical and electrochemical perturbations.

### 8.3. Integrated Soft Energy Systems: From Materials to Devices

A defining advantage of LM composites is their compatibility with additive manufacturing and multifunctional integration. Unlike conventional rigid systems that rely on discrete assembly processes, LM composites can be fabricated into monolithic and deformable architectures that integrate sensing, heating, energy storage, and electrical conduction within a single material platform. At the materials level, printable LM composites have enabled multifunctional soft structures capable of combining conductive, electrochemical, and thermal functionalities [51,94]. In particular, Ga–CB–SIS composites have been used to fabricate integrated sensor–heater–battery systems, where polymer-assisted rheology and adhesion enable stable and deformable multifunctional devices [63]. An example of such integrated soft energy devices is presented in Figure 8E.

At the device level, multilayer architectures further demonstrate the importance of binder-controlled integration. In 3R battery systems, SIS-based composites are employed across multiple printed layers to provide cohesion, elasticity, and interfacial adhesion while enabling room-temperature fabrication without sintering [44]. More broadly, multimaterial additive manufacturing approaches for LM electronics show that interfacial compatibility between conductive and insulating layers is essential for maintaining device stability during deformation [108,109]. These observations are consistent with broader advances in printable LM electronics. Polymer-functionalized LM systems and biphasic LM composites enable sinter-free and stretchable conductive architectures, demonstrating that device-level functionality arises from matrix-controlled rheology, interfacial stabilization, and structural cohesion rather than from the metallic phase alone [79].

### 8.4. Positioning Relative to Other Soft Energy Strategies

Compared with conventional soft energy approaches—such as gel electrolytes, serpentine interconnects, kirigami structures, and intrinsically stretchable polymers—LM composites represent a distinct material design strategy. Traditional systems accommodate deformation primarily through geometric or structural engineering, whereas LM systems rely on intrinsic material adaptability enabled by fluidity, dynamic interfaces, and reconfigurable conductive pathways [1,111]. However, this same adaptability also introduces new challenges. The fluidic nature of LM phases can promote leakage, droplet coalescence, and continuous interfacial evolution, potentially destabilizing electrochemical performance. These effects must therefore be regulated through binder and matrix engineering, which stabilize phase behavior, maintain interfacial integrity, and preserve conductive pathways during deformation and cycling [83,112]. Consequently, LM-based systems shift the design philosophy of soft energy storage away from purely structural engineering and toward interfacial and composite engineering, where binder selection becomes a central control parameter governing mechanical reliability, transport stability, and electrochemical functionality. Overall, the performance of LM composites depends strongly on how effectively the polymer matrix stabilizes the liquid metal phase during deformation, interfacial evolution, and electrochemical cycling. While LM phases provide intrinsic deformability and conductive behavior, polymer binders determine whether these properties can be maintained under practical operating conditions. This combination of conductivity, deformability, and matrix stabilization has enabled the development of increasingly robust and multifunctional soft energy-storage systems [116].

To facilitate comparison among representative LM–binder systems reported in the literature, Table 2 summarizes key quantitative performance metrics, including electrical performance, stretchability, cycling stability, self-healing capability, recyclability, and processing approaches. The comparison highlights the diversity of binder-enabled design strategies and illustrates how different binder architectures influence the electro–chemo–mechanical performance of LM composites across various soft energy storage and electronic applications. As shown in Table 2, no single binder system simultaneously maximizes conductivity, stretchability, cycling stability, self-healing behavior, and sustainability. Instead, binder selection requires balancing multiple performance requirements depending on the target application. Elastomeric binders generally favor deformability and printability, whereas multifunctional dynamic polymer networks provide improved interfacial stability, damage tolerance, and electrochemical durability. These observations further support the central premise of this review that binder design is a key factor governing the overall performance of LM composite systems. However, improvements in one performance attribute do not necessarily translate to improvements in all others.

While stronger binder networks generally improve droplet stabilization and mechanical integrity, excessive confinement may reduce liquid metal mobility, limit self-reconfiguration, and increase processing complexity. Consequently, binder selection often involves balancing stability, conductivity, deformability, manufacturability, and long-term durability rather than maximizing a single performance metric.

## 9. Sustainability and Scalable Manufacturing

The long-term viability of LM composites for soft energy storage depends not only on electrochemical and mechanical performance, but also on the sustainability, scalability, and manufacturability of the underlying material systems. While Ga-based LMs provide intrinsic conductivity, deformability, and reconfigurable interfaces, these advantages can only be translated into practical technologies when combined with binder systems capable of regulating rheology, stabilizing interfaces, enabling low-temperature processing, and supporting material recovery. Consequently, sustainability in LM-based soft energy systems is fundamentally linked to binder-centered composite engineering rather than to the metallic phase alone.

Compared with conventional metallic electrode fabrication, LM composites offer several inherent advantages for sustainable manufacturing. Many LM-based systems can be processed under ambient or near-room-temperature conditions without high-temperature sintering, vacuum deposition, or rigid assembly procedures, thereby reducing thermal processing requirements and improving compatibility with flexible and heat-sensitive substrates. Recent advances in printable LM composites further demonstrate that polymer-assisted rheological regulation enables additive manufacturing routes such as direct ink writing, extrusion printing, and multimaterial deposition while minimizing process complexity and material waste [117,118]. In these systems, the binder acts as a manufacturing-enabling matrix that transforms intrinsically unstable LMs into processable and structurally stable functional materials.

### 9.1. Energy-Efficient and Low-Temperature Manufacturing

One of the most significant advantages of LM composites is their compatibility with low-temperature and sinter-free fabrication. Conventional metallic electrodes often require thermal annealing, solvent-intensive processing, or rigid lamination procedures to achieve sufficient conductivity and adhesion. In contrast, polymer-assisted LM systems can be printed, patterned, and integrated at room temperature while maintaining electrical continuity and mechanical compliance.

Recent studies on printable LM elastomer composites demonstrated that viscoelastic polymer matrices enable controlled extrusion, rapid shape retention, and stable conductive architectures without post-sinter thermal treatment [50,79,107]. Similar room-temperature additive manufacturing strategies have been employed in stretchable LM electronics and soft battery architectures, where binder-controlled rheology and interfacial adhesion support scalable fabrication on deformable substrates [44,51]. These approaches reduce manufacturing energy demand while improving compatibility with wearable and biointegrated systems.

Importantly, the sustainability benefits of low-temperature processing are closely tied to binder functionality. Without polymer-mediated stabilization, LM droplets undergo uncontrolled spreading, coalescence, and phase separation, preventing reliable printing and multilayer integration. Polymer design therefore strongly influences whether LM systems can be processed through scalable and energy-efficient fabrication routes.

### 9.2. Recyclability and Circular Material Design

Beyond manufacturing efficiency, recyclability and material reusability are becoming increasingly important for soft and wearable electronics due to growing concerns regarding electronic waste and resource consumption. Dynamic polymer binders and reconfigurable LM composites offer unique opportunities for repairable, reprocessable, and recyclable soft energy systems.

Unlike permanently crosslinked thermoset systems, dynamic elastomeric binders can enable reversible adhesion, network rearrangement, and solvent-assisted material recovery. Recent studies on recyclable LM composites demonstrated that elastomeric polymer matrices can support repeated deformation and reconfiguration while preserving conductive functionality and structural integrity [51,94]. Related dynamic polymer systems further showed that reversible intermolecular interactions can facilitate mechanical repair and material reprocessing, supporting recyclable and reusable device designs in deformable electronics [119]. Ga recovery and phase recyclability also represent important advantages of LM systems. Because the metallic phase remains fluidic, LM domains can potentially be separated, redistributed, or reprocessed more readily than fractured rigid conductive fillers. This potential for material recovery and reuse represents an important advantage of LM-based systems compared with many conventional metallic components, which are often more difficult to separate, reprocess, or recycle after device failure or end-of-life operation. From a broader sustainability perspective, solvent selection and end-of-life management also require consideration during LM composite design. The use of water-processable or low-toxicity binder systems can reduce the environmental burden associated with electrode fabrication and solvent handling. Furthermore, the recoverability of liquid metal phases may facilitate material separation, Ga recovery, and component reuse at the end of device life, thereby supporting more circular and resource-efficient manufacturing strategies. These considerations highlight the importance of integrating environmental impact and lifecycle management alongside electrochemical and mechanical performance when developing next-generation LM-based energy systems [51,94,119,120]. In multifunctional LM composites, binder-assisted phase confinement and reversible interfaces therefore provide pathways toward repairable and recyclable soft energy architectures rather than disposable devices.

### 9.3. Scalable Additive Manufacturing and Printing

Scalable fabrication of LM-based soft energy systems increasingly relies on additive and digitally programmable manufacturing approaches. Polymer-assisted LM inks have demonstrated compatibility with extrusion printing, direct ink writing, aerosol printing, and multimaterial additive manufacturing techniques, enabling complex deformable architectures while reducing material waste relative to conventional subtractive manufacturing.

Recent studies on printable LM systems showed that rheologically optimized polymer matrices enable stable droplet dispersion, shear-thinning behavior, and interlayer adhesion during multilayer printing [18,79,80,81]. In multimaterial LM electronics, binder-controlled interfacial compatibility allows conductive, insulating, sensing, and electrochemical layers to be integrated into cohesive soft device architectures [108,109]. Similar approaches have been applied to stretchable batteries and multifunctional LM composites, where polymer-assisted printability enables simultaneous integration of energy storage, sensing, and thermal management functions [42,43,63]. Importantly, scalable manufacturing depends not only on printability but also on long-term rheological and structural stability. Binder composition determines whether LM inks maintain homogeneous dispersion during storage, extrusion, and deposition while preventing droplet aggregation and phase segregation. Consequently, the scalability of LM manufacturing is fundamentally governed by binder-mediated regulation of rheology, interfacial stabilization, and structural cohesion.

### 9.4. Challenges in Industrial Translation

Despite promising laboratory-scale demonstrations, several barriers remain for industrial translation of LM-based soft energy systems. A major challenge is the long-term stability of printable LM inks, since droplet coalescence, oxidation, sedimentation, and rheological drift can compromise storage stability, print reproducibility, and device uniformity. Recent studies on printable LM composite inks emphasize that stable rheology, oxide management, and particle–matrix interactions are critical for reliable large-area manufacturing [121,122,123].

Another challenge arises from the intrinsically dynamic nature of LM interfaces. Oxidation-induced changes in surface chemistry, electrolyte interactions, and interfacial tension can generate variability in electrochemical and mechanical behavior during processing and operation. Recent work on oxidation-dominated rheology further shows that ambient oxidation can strongly alter LM flow behavior and interfacial properties, underscoring the need for binder systems that regulate oxide formation and stabilize LM interfaces during manufacturing [124].

Industrial translation also requires scalable patterning and integration strategies. Although direct ink writing, extrusion printing, laser processing, and multimaterial additive manufacturing have advanced rapidly, reliable scale-up still requires control over printing resolution, interlayer adhesion, encapsulation, and compatibility with flexible substrates. Recent reviews of LM-based flexible electronics highlight that fabrication, integration, stability, and reliability remain central barriers to commercialization [125]. Finally, practical deployment in wearable and biointegrated energy systems requires reliable encapsulation, low-leakage operation, mechanical durability, and standardized quality control. In addition, the relatively high cost of Ga-based LMs may limit large-scale adoption in cost-sensitive applications. Long-term environmental stability also remains a concern, as LM phases can undergo corrosion, oxidation, and humidity-induced interfacial degradation that may affect device reliability and lifetime [120]. Future scalable manufacturing will therefore depend strongly on polymer binders that stabilize LM dispersions, maintain rheological consistency, suppress interfacial degradation, and support reliable multilayer fabrication.

### 9.5. Practical Binder Selection Guidelines

Based on the literature discussed throughout this review, binder selection should be guided by the specific performance requirements of the target application. Because no single binder architecture simultaneously optimizes all performance metrics, rational binder design requires balancing mechanical, electrochemical, manufacturing, and sustainability considerations. Table 3 summarizes general binder-selection guidelines for representative design objectives in LM composite systems. These guidelines highlight that binder selection is inherently application-dependent and should be based on the desired balance between stability, conductivity, deformability, manufacturability, self-healing capability, and sustainability.

## 10. Challenges and Future Perspectives

Despite rapid advances in LM composite systems, binder engineering remains limited by the lack of standardized evaluation methods, incomplete mechanistic understanding, and insufficiently predictive design frameworks. While recent studies have demonstrated that binders actively regulate droplet stability, interfacial chemistry, rheological behavior, and electrochemical performance, the field still relies heavily on empirical optimization rather than quantitatively guided materials design.

One of the most significant challenges is the absence of unified metrics for evaluating coupled electro–chemo–mechanical performance. Current studies frequently report electrochemical, rheological, and mechanical properties independently, making it difficult to compare material systems under realistic operating conditions involving simultaneous deformation and electrochemical cycling. This issue is broadly recognized in soft energy storage research, where standardized benchmarking protocols for deformable energy devices remain underdeveloped [1]. Future progress will require integrated testing methodologies capable of correlating conductivity retention, interfacial stability, rheological response, cycling durability, and mechanical resilience under dynamically coupled conditions.

Another major limitation is the insufficient understanding of dynamic LM interfaces. In LM-based systems, electrochemical behavior is strongly influenced by continuously evolving interfacial phenomena, including oxide growth and rupture, electrocapillary effects, wetting transitions, ion adsorption, and compositional redistribution [15,72,100,101]. These processes directly influence charge transfer behavior, droplet morphology, and long-term electrochemical stability. Recent studies further demonstrate that oxidation-controlled interfacial dynamics can strongly alter LM rheology, wettability, and electrochemical accessibility during operation [124]. Developing binder systems capable of stabilizing these evolving interfaces during simultaneous deformation and electrochemical cycling therefore remains a central challenge for practical LM energy systems.

Design trade-offs also remain largely unresolved. Binder systems must simultaneously balance mechanical compliance, conductive stability, droplet confinement, interfacial compatibility, ionic accessibility, printability, and dynamic adaptability. However, improving one property often compromises another. For example, increasing crosslink density may improve structural integrity while reducing stretchability or ion transport, whereas highly compliant matrices may weaken conductive percolation stability. Reviews of multifunctional battery binders consistently identify such competing requirements as a major barrier to rational materials optimization [46,47]. Addressing this issue will require multiscale design strategies that couple molecular-level interactions with mesoscale transport behavior and device-level mechanical performance.

A further challenge is the continued reliance on empirical formulation approaches. Many LM composite systems are still developed through trial-and-error optimization of polymer composition, filler loading, solvent systems, and processing conditions. Predictive understanding of how binder chemistry governs LM interfacial behavior, rheology, conductive network evolution, and electrochemical functionality remains limited. Future progress will therefore depend on establishing quantitative structure–property relationships supported by advanced characterization techniques, in situ electrochemical analysis, and multiscale computational modeling. In particular, operando characterization methods capable of simultaneously probing interfacial chemistry, droplet dynamics, and conductive network evolution under deformation are expected to become increasingly important.

Scalable manufacturing and long-term reliability also present major challenges. Although additive manufacturing and direct-printing approaches have enabled rapid progress in deformable LM devices, industrial translation still requires improvements in ink stability, reproducibility, encapsulation reliability, oxidation control, and multilayer integration. Recent studies on printable LM electronics highlight that rheological drift, droplet coalescence, and interfacial degradation can significantly compromise manufacturing consistency and device durability [125]. Future binder systems must therefore simultaneously optimize processability, storage stability, environmental resistance, and interfacial robustness to enable scalable and commercially viable manufacturing.

Looking forward, binder engineering presents opportunities for tunable and adaptive material systems in which viscoelasticity, interfacial interactions, transport behavior, and self-healing functionality can be systematically tuned for specific applications. Dynamic polymer networks, supramolecular interactions, multifunctional fillers, and reconfigurable LM architectures may enable next-generation soft energy systems improved adaptability and damage tolerance. In addition, emerging data-driven materials design strategies, machine learning-assisted formulation optimization, and high-throughput rheological screening could accelerate discovery of multifunctional binder systems with tailored electro–chemo–mechanical properties. Beyond soft batteries and supercapacitors, these concepts may also extend to biointegrated electronics, self-powered wearable systems, soft robotics, and multifunctional human–machine interfaces, where materials must simultaneously maintain conductivity, interfacial stability, and mechanical compliance under complex operating conditions. In these applications, binder design is expected to play an increasingly important role in regulating interfacial behavior, mechanical adaptability, and long-term device stability.

Overall, future progress in LM composites will depend on developing polymer binders with improved interfacial stability, rheological control, and long-term electrochemical reliability. Achieving this goal will require stronger connections between molecular-scale interactions, interfacial chemistry, processing behavior, and device-level performance. Such advances are expected to support the development of more reliable, scalable, and multifunctional LM-based soft energy systems.

## 11. Conclusions

Liquid metal (LM) composites have emerged as a fundamentally distinct materials platform for soft energy storage, combining metallic conductivity with fluidic deformability and dynamically evolving interfaces. These unique characteristics enable mechanical adaptability and multifunctionality; however, they also introduce critical challenges—including droplet coalescence, leakage, interfacial instability, and transport heterogeneity—that cannot be resolved by the LM phase alone. This Review establishes that the binder functions as the central regulator of LM composite behavior, governing droplet stabilization, conductive network integrity, interfacial compatibility, rheological response, transport stability, and processability across multiple length scales. Throughout diverse LM systems and applications, the electro-chemo-mechanical performance of the composite consistently depends on how effectively the binder mediates interactions between the LM, active materials, electrolytes, and surrounding matrix. In this context, the binder should be regarded not simply as a passive structural additive, but as an active component that strongly influences the stability and functionality of LM-based energy systems.

Future advances in LM-enabled soft energy storage will therefore depend on the rational development of multifunctional binder systems capable of integrating mechanical adaptability, interfacial regulation, dynamic transport control, self-healing capability, and scalable manufacturability within unified material architectures. Emerging opportunities in programmable polymer networks, adaptive interfaces, and multimaterial additive manufacturing may further enable LM composites with tunable electro-chemo-mechanical behavior for specific applications.

By shifting the design paradigm from passive structural support toward active binder engineering, LM composites can evolve from proof-of-concept materials into more reliable and scalable platforms for wearable, deformable, and biointegrated electronics. More broadly, this binder-centered perspective highlights the importance of interfacial and composite engineering for developing soft energy systems that combine electrochemical functionality with mechanical resilience and manufacturability.

## Figures and Tables

**Table 1:** Functional spectrum of binders from passive to active behavior in conventional and liquid metal-based electrodes.

Binder Class	Representative Systems	Key Functionality in Electrode Systems	Functional Classification	Refs.
PVDF	Conventional cathodes; benchmark LIB binder	Provides adhesion and cohesion during slurry processing; limited interfacial adaptability and negligible contribution to transport regulation	Predominantly passive	[46,47]
CMC/SBR	Graphite and Si-containing anodes	Water-processable binder system with improved mechanical compliance and moderate accommodation of volume changes	Passive-to-semi-active	[46,53]
PAA and polysaccharide-based binders	Si and high-volume-change electrodes	Strong interfacial interactions through polar functional groups; improves adhesion, stress tolerance, and structural stability during cycling	Active (interfacial/mechanical)	[54,55,56]
Conductive or ion-transport-supporting binders	Thick electrodes; high-loading LIB systems	Couples mechanical support with ion/electron transport regulation and electrolyte accessibility	Active (transport/interfacial)	[47,57,58,59]
Dynamic/self-healing polymer binders	Damage-tolerant and high-strain electrodes	Reversible intermolecular interactions enable stress dissipation, crack repair, and structural recovery after deformation	Active (dynamic/mechanical)	[7,60,61,62]
SIS-based elastomeric binders	Stretchable LM composites; printable soft batteries; 3R battery architectures	Provides LM droplet confinement, rheological control, adhesion, and mechanical compliance while maintaining conductive pathways under deformation	Active (mechanical/structural/processability)	[43,44,63]
LM-incorporated dynamic polymer binders	LM-containing Si microparticle anodes	Dynamic noncovalent interactions couple binder mechanics with LM-enabled conductive stability and adaptive structural regulation	Highly active (electro–chemo–mechanical)	[7]
Biphasic and LM–polymer composite systems	EGaIn–Ag–SIS; LM elastomer composites; printable conductors	Hybrid conductive networks stabilize LM dispersion, suppress coalescence, and enable sinter-free stretchable electronics	Highly active (structural + conductive coupling)	[48,49,50]
Reconfigurable and recyclable LM composite binders	3R systems; repairable soft electronics	Enables reversible adhesion, reconfiguration, repairability, and recyclable device architectures through dynamic polymer networks	Highly active (dynamic + lifecycle regulation)	[44,49,51,52]

**Table 2:** Quantitative comparison of representative binder-enabled liquid metal composite systems, including electrical performance, stretchability, cycling stability, self-healing capability, recyclability, and processing methods.

Binder/Matrix System	LM Type	Application	Electrical Performance	Stretchability	Cycling Stability/Durability	Self-Healing/Recyclability	Processing Method	Ref.
Noncovalent LM-incorporated polymer binder (PAA/CNF/EGaIn)	EGaIn	Si anode (LIB)	ICE = 87.9%; 1787.9 mAh g^−1^ at 1C	Not reported	Stable over 300 cycles at 1C; full cell retained 1.9 mAh cm^−2^ after 100 cycles	Dynamic LM-assisted crack repair and electrical reconnection	Conventional slurry electrode fabrication	[7]
Ga–CB–SIS composite	Ga	Stretchable battery anode	Conductive printable electrode; R ≈ 4 Ω	100% strain tolerance	Maintained performance during repeated deformation	Self-healing; recyclable with ≈98% Ga recovery	Digital printing/3D printing	[63]
Foam Ag_2_O–Ga battery	Ga	Stretchable battery	Areal capacity = 40.91 mAh cm^−2^; specific capacity = 221.16 mAh g^−1^	50% strain	≈5-fold improvement in charge–discharge cycling	Ga-enabled self-adaptation	3D printed foam electrodes	[43]
3R Ag_2_O–Ga battery	Ga	Stretchable battery	Areal capacity = 26.37 mAh cm^−2^; increased to 30.32 mAh cm^−2^ after 10 cycles at 100% strain	≈200% strain	Capacity maintained under cyclic stretching	Repairable and recyclable battery architecture	Fully 3D printed	[44]
EGaIn–Ag–SIS biphasic composite	EGaIn	Stretchable conductor/current collector	EGaIn conductivity = 3.4 × 10^6^ S m^−1^	>600% strain	Stable resistance under repeated deformation	Intrinsic LM-assisted damage tolerance	Direct writing/printing	[50]
EGaIn–Ni–SIS composite	EGaIn	Recyclable stretchable electronics	Initial resistance = 0.41 Ω; GF ≈ 0.39 at 100% strain	100% strain	Stable electromechanical response	LM recovery ≈ 99.5%	Digital printing	[49]
Ag-coated LM/SIS composite	Ga-based LM	EMI shielding/stretchable electronics	EMI shielding effectiveness > 75 dB	200% strain	Maintained shielding under deformation	Recyclable composite architecture	Printable composite	[48]
LM embedded elastomer (LMEE)	EGaIn	Soft electrical/thermal devices	Conductivity = 5 × 10^4^ S cm^−1^ at 80 vol% LM	Highly compliant elastomer	Stable after activation and repeated use	Not reported	Direct Ink Writing (DIW)	[81]
SIS-based LM self-healing composite	EGaIn	Reconfigurable soft electronics	Conductivity up to 45,400 S cm^−1^ under strain	1200% strain	Multiple damage events tolerated	Self-healing, reconfigurable, recyclable	Embossing/soft lithography	[94]

**Table 3:** General design guidelines for binder selection in liquid metal composite systems, highlighting the relationships between target performance requirements and desirable binder characteristics.

Design Objective	Desired Binder Characteristics
Droplet stabilization	Strong interfacial adhesion, effective confinement, resistance to coalescence
Printability	Shear-thinning rheology, viscoelastic recovery, stable LM dispersion
High conductivity	Minimal transport resistance, maintenance of conductive percolation pathways
Stretchability	Elastomeric matrix, low modulus, strain accommodation capability
Self-healing	Dynamic covalent bonds, supramolecular interactions, reversible network formation
Cycling stability	Stable electrode–electrolyte interfaces, resistance to interfacial degradation
Sustainability	Recyclable polymers, low-toxicity processing, support for material recovery

## Data Availability

No new data were created or analyzed in this study. Data sharing is not applicable to this article.
